# Spontaneous Abortion and Preterm Labor and Delivery in Nonhuman Primates: Evidence from a Captive Colony of Chimpanzees (*Pan troglodytes*)

**DOI:** 10.1371/journal.pone.0024509

**Published:** 2011-09-16

**Authors:** Derek E. Wildman, Monica Uddin, Roberto Romero, Juan M. Gonzalez, Nandor Gabor Than, Jim Murphy, Zhuo-Cheng Hou, Jo Fritz

**Affiliations:** 1 Center for Molecular Medicine and Genetics, Wayne State University School of Medicine, Detroit, Michigan, United States of America; 2 Department of Obstetrics and Gynecology, Wayne State University School of Medicine, Detroit, Michigan, United States of America; 3 Perinatology Research Branch, Eunice Kennedy Shriver National Institute of Child Health and Human Development, National Institutes of Health, Department of Health and Human Services, Detroit, Michigan, United States of America; 4 Department of Epidemiology, University of Michigan School of Public Health, Ann Arbor, Michigan, United States of America; 5 Primate Foundation of Arizona, Mesa, Arizona, United States of America; University of Delaware, United States of America

## Abstract

**Background:**

Preterm birth is a leading cause of perinatal mortality, yet the evolutionary history of this obstetrical syndrome is largely unknown in nonhuman primate species.

**Methodology/Principal Findings:**

We examined the length of gestation during pregnancies that occurred in a captive chimpanzee colony by inspecting veterinary and behavioral records spanning a total of thirty years. Upon examination of these records we were able to confidently estimate gestation length for 93 of the 97 (96%) pregnancies recorded at the colony. In total, 78 singleton gestations resulted in live birth, and from these pregnancies we estimated the mean gestation length of normal chimpanzee pregnancies to be 228 days, a finding consistent with other published reports. We also calculated that the range of gestation in normal chimpanzee pregnancies is approximately forty days. Of the remaining fifteen pregnancies, only one of the offspring survived, suggesting viability for chimpanzees requires a gestation of approximately 200 days. These fifteen pregnancies constitute spontaneous abortions and preterm deliveries, for which the upper gestational age limit was defined as 2 SD from the mean length of gestation (208 days).

**Conclusions/Significance:**

The present study documents that preterm birth occurred within our study population of captive chimpanzees. As in humans, pregnancy loss is not uncommon in chimpanzees, In addition, our findings indicate that both humans and chimpanzees show a similar range of normal variation in gestation length, suggesting this was the case at the time of their last common ancestor (LCA). Nevertheless, our data suggest that whereas chimpanzees' normal gestation length is ∼20–30 days after reaching viability, humans' normal gestation length is approximately 50 days beyond the estimated date of viability without medical intervention. Future research using a comparative evolutionary framework should help to clarify the extent to which mechanisms at work in normal and preterm parturition are shared in these species.

## Introduction

Sporadic pregnancy loss in humans is a common event. Approximately 70% of human conceptions fail to achieve viability. Between 22 and 50% are lost before the first missed menstrual period [1], [2], and maternal stress among other causes can play a role in pregnancy loss [3]. In pregnancies that are clinically recognized before 20 weeks of gestation from the last menstrual period, loss occurs in approximately 15% [4]. The causes include genetic abnormalities, hormonal/metabolic disorders, uterine anatomical abnormalities, infectious causes, environmental factors, thrombophilia, autoimmune disorders, and others which remain unexplained [5]. Spontaneous human preterm birth remains an enigma and the major unsolved problem in modern obstetrics and perinatal medicine [6]. The preterm parturition syndrome in which human labor and delivery are premature (i.e. between 20 to 37 weeks of gestation) affects one of every eight human pregnancies in the United States at an annual cost of $26 billion per year [7], [8], [9], [10]. Prematurity remains the leading cause of perinatal [11] and infant [12] morbidity and mortality. In humans, the preterm parturition syndrome is caused by multiple pathological processes, which can lead to the activation of the common pathway. Examples of these pathological processes include among others: infection, uterine hemorrhage, uterine overdistention, cervical disease, abnormal allograft reaction, and endocrine disorders [13]. Despite strong efforts in perinatal research, the incidence of preterm birth continues to rise [14]. That preterm birth is so prevalent in human populations raises the question whether, in certain environments, an evolutionary advantage could possibly be gained by delivering a fetus prematurely. The maternal adaptation of preterm birth could be of evolutionary advantage in the face of poor intrauterine and environmental conditions to limit the energetic cost of the pregnancy [15]. In order to assess this concept it is necessary to take a comparative evolutionary approach by examining the rate of pregnancy loss in nonhuman primates and other mammals.

**Figure 1:**
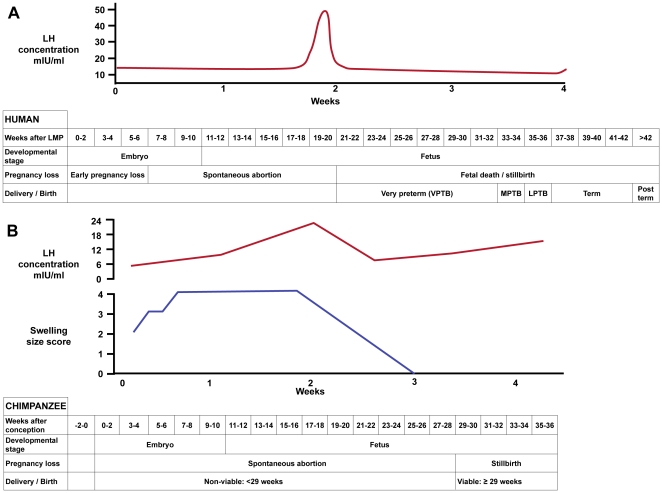
Comparison of gestation in humans (A) and chimpanzees (B). A typical 40 week human gestation is measured as beginning (week 0) at the onset of the last menstrual period (LMP) while gestation in chimpanzees is measured as beginning at roughly the time of ovulation (week 0). Graphs A and B show a typical ovulatory cycle, with typical patterns of serum luteinizing hormone (LH) concentrations (red lines) in human [68] and chimpanzee [69] as well as chimpanzee swelling size score (blue line) [69]. Tables (A human; B chimpanzee) compare gestational parameters and different categories of pregnancy complications related to gestational length. VPTB: very preterm birth; MPTB: moderately preterm birth; LPTB: late preterm birth [70].

In the present study we sought to examine the length of gestation in one of humankind's closest relatives, the chimpanzee (*Pan troglodytes*). Molecular phylogenetic analyses indicate that humans and chimpanzees are sister taxa to the exclusion of gorillas and the more distantly related orangutans [16], [17], and analysis of the chimpanzee genome indicates that the two species are genetically similar, sharing 98.7% sequence identity [18]. Despite these similarities, there are clear differences between human and chimpanzee pregnancies. The mean length of gestation in common chimpanzees has been reported as 227 days compared to 280 in humans [19], [20]. This 53-day difference in gestation length is somewhat misleading. Human gestation length is commonly measured from the first day of the last menses, about two weeks before conception. In contrast, chimpanzee gestations are measured from the last day of maximal sex skin tumescence during a cycle in which copulation was observed, which correlates roughly to the point at which ovulation occurs in the chimpanzee menstrual cycle (Fig. 1). Therefore, the actual difference in gestation length between human and common chimpanzees is less than 40 days [21]. Furthermore, natural selection during human evolution has resulted in anatomical changes including the expansion of the cranium associated with encephalization and the remodeling of the pelvis during the emergence of bipedalism that may have affected parturition [22], [23]. These anatomical changes that resulted in a relatively larger fetal head and smaller birth canal may have necessitated adjustments underlying the labor and birth process (i.e. parturition has a longer duration in humans than in chimpanzees). Indeed, the duration of parturition in humans is, on average, longer than the 80-minute duration that has previously been observed in chimpanzees [20]. The genetic similarities shared between humans and chimpanzees contrasted with their gross anatomical and reproductive differences make chimpanzees an attractive comparative taxon for examining the evolution of human pregnancy and parturition.

In the present study we took advantage of historical records of chimpanzee gestation length in pregnancies that occurred at the Primate Foundation of Arizona. Cumulatively, we were able to study monthly menstrual cycle charts from chimpanzees housed at this facility over a period of 30 years. The historical records allow us to 1) confirm previous reports regarding the length of normal gestations in chimpanzees; 2) identify the pregnancies that failed due to spontaneous abortion; and 3) reveal whether preterm delivery actually occurs in this species. These observations represent the first systematic investigation of preterm delivery in chimpanzees.

## Materials and Methods

### Ethics Statement

The data collection for this study consisted entirely of a review of existing longitudinal records at the Primate Foundation of Arizona (PFA). There was no animal contact.

The Primate Foundation of Arizona is a private, non-profit, biomedical research facility accredited by the Association for Assessment and Accreditation of Laboratory Animal Care International. The data collection protocol was reviewed and approved by the PFA Institutional Animal Care and Use Committee (permit # 52107) and adhered to the legal requirements of the United States Department of Agriculture.

### Observation of Chimpanzees

**Figure 2:**
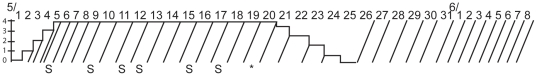
Idealized chimpanzee cycle chart. The shown partial chart spans the dates from May 01 (5/1) to June 08 (6/8) and indicates a month in which a conception occurred. The Y-axis indicates degree of swelling on a scale from 0–4, in which 0 = no swelling, and 4 = maximum swelling. Conventionally, a diagonal line is drawn each day from the point of observation. If swelling was observed, a horizontal line is drawn on that day reflecting the amount of observed swelling. Diagonal lines are drawn on each day in order to indicate a measurement was made. Shown is an example in which the anogenital swelling phase begins on day two in the month of May, reaches maximal height on day 5, stays maximal until day 20, and is no longer swollen on day 25. Days in which copulations occurred are depicted by the first initial of the male consort, in this example  =  S. Ovulation occurs at some point during the maximally swollen phase, and if the chimpanzee becomes pregnant, the conception is estimated to have occurred on the last day of maximal swelling (as indicated by asterisk = * on 5/20).

Chimpanzees housed at the PFA were observed daily over a period of thirty years. Daily notations of sex skin swelling were recorded on cycle charts [24] for each individual female (Fig. 2). Menstruation, all observed copulations and delivery dates were noted on cycle charts.

### Determination of length of gestation

Conception in chimpanzee pregnancies was estimated as the date of maximal sexual skin tumescence during an ovarian cycle in which copulation was observed (n = 88). If no copulation was observed (n = 5), conception was estimated by counting backwards from delivery until the cycle of appropriate length (i.e. the closest cycle to 227 days).

### Statistical and demographic analyses

Cycle charts from all female chimpanzees of breeding age (n = 67) housed at PFA between the periods of 1969 and 2007 were examined. Each chart was examined to determine whether a pregnancy was observed, and the dates of conception and delivery were tabulated for each pregnancy. Data were excluded from analyses if the individual was shipped away from or to the PFA while pregnant and if chemical abortion was induced. In a few cases, chimpanzees became pregnant at the PFA, but were moved to New York and were subsequently housed at the Laboratory for Experimental Medicine and Surgery in Primates (LEMSIP) during their pregnancy. Similarly, three pregnancies began at LEMSIP, but the births occurred at the PFA. The pregnancies that occurred at LEMSIP, either wholly or in part, were excluded from statistical analyses, as were an additional nine pregnancies from which gestation length was not clearly estimated in the cycle chart records. When possible, relevant clinical data was consulted to examine the etiology of pregnancy failure. All included pregnancies were calculated a gestation length, and maternal age at time of pregnancy was also noted. Gestations were divided post hoc into two categories: 1) spontaneous abortion and preterm parturition (<208 days) and 2) normal gestation (208≥249 days). Fig. 1 compares reproductive parameters in humans and chimpanzees. Summary and descriptive statistics were calculated for all included chimpanzee pregnancies. A modified Shapiro-Wilk's *W* test [25] was used to test whether the distribution of gestation lengths was normal.

## Results

### Pregnancies at a captive colony of chimpanzees

This study includes data from 93 pregnancies at the Primate Foundation of Arizona between 1969 and 1999. Thirty-five females were pregnant at least once at the PFA. The earliest recorded pregnancy occurred in 1969–1970, and the last pregnancy occurred in 1999. In this latter case the pregnancy was terminated by chemical abortion due to an NIH moratorium on breeding chimpanzees for research purposes [26]. Two other pregnancies were terminated by chemical abortion for the same reason, and these pregnancies were not included in statistical analyses. In all cases, the pregnancies were terminated very early in gestation as soon as pregnancy was detected by the presence of chorionic gonadatropin in urine. The date of the last chimpanzee birth at the PFA was on 11/14/1997. This chimpanzee is now housed in a colony in Bastrop, TX USA.

### Gestation Length at PFA

**Figure 3:**
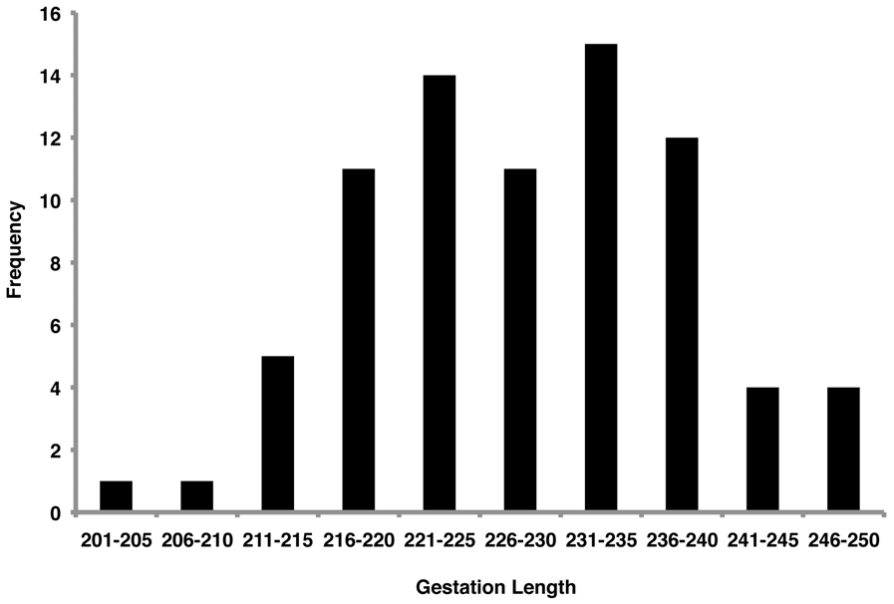
Normal variation in chimpanzee gestation. Histogram indicates the number of pregnancies per length of gestation. The frequency of singleton pregnancies that resulted in live birth is shown. These data are consistent with a normal distribution.

The estimated length of gestation in non-excluded pregnancies at PFA ranged from 39 to 249 days. When only live births from singleton pregnancies (n = 78) were included, the mean gestation length for pregnancies was estimated to be 228 days (S.D. = 9.9 days). For five of the singleton pregnancies, no copulation was observed, making determination of conception difficult. When these pregnancies were excluded, the mean gestation length remained 228 days. The frequency of singleton pregnancies that resulted in live birth is shown in Fig. 3. The data shown in Fig. 3 are consistent with the normal distribution (Shapiro-Wilk Normality Test; p = 0.78); A Pearson's Correlation Test found no significant relationship between maternal age during pregnancy and gestation length (n = 71; p = 0.43), although maternal age for all mothers was not recorded.

### Perinatal death in preterm pregnancies

Two chimpanzees housed at PFA were unable to carry infants to term and had recurrent pregnancy losses. In one case, a female had three pregnancies in which pregnancy loss was estimated to have occurred at 119, 160, and 164 days, respectively. Another female also had three pregnancies resulting in pregnancy loss with gestations estimated to last 92, 191, and 192 days, and the latter two pregnancies were recorded as resulting in stillbirths. This animal underwent a tubal ligation after it became apparent she could not carry pregnancies to term. One chimpanzee had a pregnancy that lasted only an estimated 39 days. Veterinary records estimate the age of the fetus as between 25 and 60 days. This female never became pregnant again. Of the eight other female chimpanzees that had pregnancies resulting in estimated abortions and/or preterm delivery, all had at least one successful pregnancy.

### Outcomes of offspring delivered preterm

All but one chimpanzee with an estimated gestation length≤208 days was aborted, stillborn, or died within a few days of birth. Of these seventeen offspring, only three were recorded as live births. A female chimpanzee gave birth to twins after an estimated gestation of 164 days (23 weeks, 4 days) and both twins died within four hours of delivery. Only one chimpanzee (born on 5/22/1979) with an estimated gestation length <208 days lived to adulthood, and this chimpanzee remained housed at the PFA. This chimpanzee is blind because she did not develop pupils. However, the veterinary records and cycle charts indicate two estimated dates of conception (9/27/1978 and 11/02/1978) for this individual, thus ambiguity regarding gestation length exists for this pregnancy. If the latter date is used, this pregnancy is estimated to have been 201 days long, while the latter estimate would have resulted in a gestation of 238 days.

### Perinatal death in term pregnancies

In addition to premature fetal loss, eight additional cases of perinatal death occurred after term pregnancies. In some cases, the cause of death was not determined. In other cases the etiology was infectious and septicemia and viral pneumonia were recorded as causes of death. A chimpanzee gave birth to an infant after an estimated gestation of 210 days, and this infant died of meningitis four days after birth. Additionally, one infant died due to meconium aspiration.

## Discussion

It is well appreciated that human and chimpanzee pregnancies are similar, and it is also known that the reproductive tracts and placentas of the two species share similarities with one another [27]. Despite the resemblances, humans and chimpanzees have some notable differences in reproduction. For example, unlike humans, chimpanzee ovarian cycles are accompanied by marked tumescence and swelling of the anogenital region [19]. Moreover, human parturition is characterized by a lengthy and protracted labor process relative to that of chimpanzees. Despite these differences there is no *a priori* reason to assume that the incidence of pregnancy loss is different in humans vs. chimpanzees. In the current study we found that in general, chimpanzee pregnancies last approximately 228 days, but that there is normal variation of approximately ±20 days surrounding this average length of gestation. In addition to normal pregnancies, there is evidence for embryonic and fetal loss in approximately 16% of the clinically recognized pregnancies that occurred at the PFA. One difficulty in estimating rates of pregnancy loss is that we were unable to detect early (i.e. preimplantation) pregnancy loss. Early pregnancy loss does not present with any clinical symptoms; instead, it appears similar to a normal menstrual cycle. It is estimated that in humans a high proportion of early pregnancy loss (∼50%) occurs in this pre-clinical period.

### Implications of Pregnancy Loss in Chimpanzees

Taken at face value, pregnancy loss is apparently detrimental to the reproductive success of a given population or species. However, natural selection does not favor maximal reproductive output because of the high fitness cost to the parents [28], [29]. Preterm delivery can be due to many causes including genetic abnormalities, hormonal/metabolic disorders, uterine anatomical abnormalities, environmental factors, thrombophilia, infectious causes, and autoimmune disorders [13]; however, intrauterine infection is the pathological process for which the most robust causal pathway has been established [30], [31], [32]. Intrauterine infection or systemic administration of microbial products to pregnant animals often results in preterm delivery [33], [34], [35], [36], although negative results have also been reported [37] In the context of intrauterine infection the onset of labor can be considered a host defense mechanism against infection. The latter process enables the mother to eliminate the infected tissue and allow the fetus to exit a hostile environment [38], [39].

The current study suggests that mechanisms for inducing premature labor predate the divergence of humans and chimpanzees. The concept of ancient origins of premature birth is further supported by experimental evidence from mice and other mammals in which inflammation and/or bacteria induce preterm delivery [33], [34], [35], [36]. Moreover, premature birth in equine, bovine and ovine species is also associated with infection [40], [41], [42], [43]. Taken together, these findings suggest that underlying mechanisms for infection induced preterm delivery have relatively ancient roots, and that searches for the molecular underpinnings of these mechanisms should not be limited to recently occurring events in human evolution. Deciphering the evolutionary tradeoffs made for idiopathic and other causes of preterm birth will likely prove to be a greater challenge.

### Did a chimpanzee born preterm survive to adulthood?

**Figure 4:**
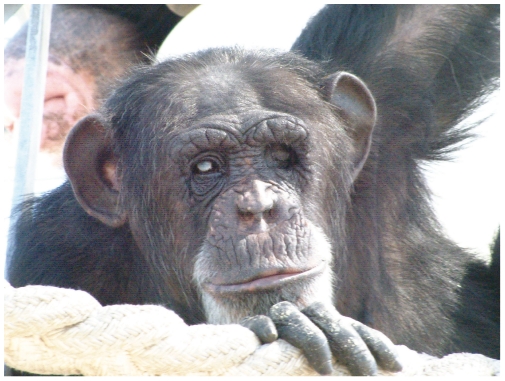
A candidate survivor of preterm delivery in chimpanzees. This photograph shows a chimpanzee that was born blind and potentially premature.

As mentioned above, all of the offspring born with an estimated gestation length of <208 days were born dead or died shortly after birth with one exception – a female chimpanzee with an estimated gestation of 201 days. This birth occurred more than two standard deviations (S.D. = 9.8 days) away from the mean gestation length among PFA chimpanzees. As previously noted, this chimpanzee was born without pupils and is therefore blind (Fig. 4). The cause of this blindness is unknown; however, medical records indicate that the mother was given a de-worming medication, at approximately 12 weeks of gestation, and it is possible that this medication played a role in the eye pathology because that is the time when pupils would have developed. However, there was another estimate of conception date made for this pregnancy, one that would have occurred approximately one month earlier. If this second conception date was indeed accurate, then the chimpanzee depicted in Fig. 4 would not have been born premature. In this case, all offspring born <208 days of gestation died shortly after birth or were stillborn. Regardless, this case points to some of the difficulties encountered when estimating gestation length in non-human primates. We also note that this chimpanzee had two successful term pregnancies of her own (est. 239 and 243 days of gestation) as well as a spontaneous abortion (est. 62 days of gestation).

### Limitations in Estimating Gestation Length in Chimpanzees

Coding cycle charts and estimating chimpanzee gestation length is an imprecise and somewhat subjective procedure. There are many reasons why an estimate may be inaccurate. First, the estimate of sexual skin swelling is qualitative not quantitative so estimating the last day of maximal skin swelling can be inaccurate and subject to inter-observer variation. Second, not all copulations that occurred were observed; therefore, the last cycle in which copulation was observed may not be the time when the chimpanzee became impregnated. Chimpanzees are known to occasionally have sex skin swellings after they become pregnant, and they do copulate during these pseudo cycles. It is difficult to observe chimpanzee menstrual blood because they fastidiously keep themselves clean. Thus, it is not practical to use the observation of menstrual blood as definitive evidence of the non-pregnant state. Because of these factors estimates of dates of conception can be off by one or more cycles. Further, modifications in housing conditions at the PFA over 30 years were not analyzed in the present study. The chimpanzees in this study were all exposed to what could be considered an artificial environment, and while some of the effects of this environment would serve to reduce the stresses associated with pregnancy (e.g. reduced metabolic stress via food provisioning, medical checkups), other factors may have increased stress (e.g. forced confinement). Despite these concerns, we feel confident that the estimates are mostly sound because we obtained a mean gestation length of 228 days, a date that is consistent with previous estimates [44]. Furthermore, the imprecision in estimations of chimpanzee conception date is biased toward reducing the number of observed preterm deliveries, and yet we were still able to confidently identify nine cases. Moreover, normal human pregnancy varies in length from 37–42 weeks. Many human conceptions are estimated based on the mother's recollection of the date of the beginning of the last menstrual cycle and this recollection is also subject to error. Ultrasound is the best means to estimate gestation length. Currently, moratoria on breeding of captive research chimpanzees at most facilities makes difficult the use of this method (or any other) of estimating gestation in humankind's closest relatives. Finally, we note that there are no estimates of the preterm delivery rate in wild chimpanzees.

### Evolutionary implications of this study

Animals have developed different strategies to balance reproductive output and fitness costs. In the primate family Hominidae [45], [46], in which humans and chimpanzees are members, this balance has focused on a strategy in which few infants are born and in which post-natal parental investment is great [47]. Indeed, the pattern of increased parental investment in offspring is a characteristic feature of all anthropoid primates – the group of primates that is made up of apes (including humans) and both Old and New World monkeys [46]. That anthropoids are characterized by relatively long gestations [48] suggests that they possess evolutionarily derived similarities in their underlying reproductive biology. The average inter-birth interval in wild chimpanzees is approximately five years [49] and it has been shown that humans with high parity rates are subject to reduced fitness [28]. The rate of preterm birth in wild chimpanzees is unknown. In some chimpanzee facilities, infants were often removed from their mothers who would then stop lactating, resume ovarian cycles, and be available for breeding in a timely fashion. However, this practice never occurred at PFA. Therefore, this aspect of the life histories of PFA chimpanzees is likely to be similar to behavior among wild born chimpanzees. It is notable that, in the one case of twins at the PFA, these twins were born prematurely and died shortly after birth. In the wild, chimpanzee twins are rare, and are known to do poorly because it is very difficult for the mother to carry and feed both infants adequately. In the best-documented case, the twins struggled, and one of the infants died of pneumonia [50]. Taken together, these observations suggest a commonality in reproductive selective pressures faced by humans, chimpanzees, and possibly other anthropoids.

The present study raises questions about the evolution of parturition in primates and other mammals. Many have considered that there must be a “trigger” that initiates parturition in mammals. Moreover, the fetus or placenta may initiate parturition at the point sufficient fetal developmental maturity necessary for survival has been reached. Searches for the “trigger” for parturition have been successful in mammal species such as sheep, in which it has been shown that progesterone stops being produced just before the onset of labor [51]. It is unclear what the causes the initiation of labor in humans and other catarrhine primates because circulating levels of progesterone do not decrease before the onset of labor. Several mechanisms have been proposed including the functional withdrawal of progesterone [52], [53], the increase of surfactant production [54], and the increase in levels of corticotropin releasing hormone [23]. However, further work is required to confirm the causal role of these proposed mechanisms.

Ellison [55] has proposed that the metaphor of a trigger for parturition may be inappropriate in the case of human parturition. Instead, he has developed what is called the crossover hypothesis. In this hypothesis it is suggested that parturition is a result of fetal growth outpacing the ability of the mother to provide sufficient resources to the developing fetus. The precise timing of the crossover of maternal supply and fetal demand results in an average gestation of 280 days in humans and 228 days in chimpanzees. Ellison further argues that crossover does not result in immediate parturition. Instead, the fetus begins to starve, and this fetal metabolic stress results in increased cortisol production. Eventually, Ellison has suggested fetal metabolic stress initiates increased arachidonic acid production in the placenta, which eventually results in increased prostaglandin production. At this tipping point uterine contractions occur and ultimately a baby is born.

There are several lines of evidence that make Ellison's crossover hypothesis attractive. First, the cervix requires several weeks to soften, ripen and dilate [56], suggesting that the preparation for labor is mediated during an ongoing process rather than by a discrete event. Moreover, there is evidence that human gestation length can be affected by metabolic demands placed on mothers. For example, during the Nazi occupation of the Netherlands birthweights decreased in association with the reduction of calories available to Dutch mothers [57]. Conversely, classic studies conducted in the Gambia showed that caloric supplement treatments given to pregnant women who normally experienced nutritional stress resulted in a reduced number of births of low birthweight infants [58].

If the crossover hypothesis is a reasonable way to understand the process of parturition in humans, it is necessary to ask when during the evolutionary past this emerged. Ellison argues that the crossover hypothesis can be explained by the massive encephalization seen in the human lineage after diverging from the chimpanzee lineage. This encephalization requires the mother to provide a relatively large supply of glucose to the developing fetal brain, delivered via the placenta during gestation and via milk during infancy and early childhood. Moreover, humans, chimpanzees, and other anthropoids all possess invasive, hemochorial placentas, and Ellison suggests that transport of glucose is facilitated by the intimate contact between fetal and maternal circulations in species with hemochorial placentation. The maximum amount of placenta glucose transport is ten times greater in humans vs. sheep, a species with a less invasive epitheliochorial placenta. Recent phylogenetic studies have conclusively demonstrated that the hemochorial form of placentation was present at the time of the last common ancestor of primates, and this placental type was also likely present at the time of the last common ancestor of placental mammals [59], [60]. However, even though the anthropoid primates all possess hemochorial placentas it is only the African ape clade (gorillas, chimpanzees, and humans) in which the trophoblast invades all the way into the inner myometrium [61]. This deep invasion has been argued to be responsible for advanced fetal brain development and lack of invasion has been associated with preeclampsia, an obstetrical syndrome also seen only in the African apes [61]. Thus, ape specific invasion of the trophoblast into the myometrium renders it anatomically plausible that the crossover hypothesis may apply to chimpanzees and gorillas, as well as to humans.

The crossover view of parturition further implies a range of variation in gestation length in association with the metabolic demands placed on the mother as well as the metabolic demands placed on the fetus. The present study demonstrates that the mean gestation length of chimpanzee pregnancy is 228 days, but our data also demonstrate a 40-day normal range of variation in chimpanzee gestations. This normal range highlights the ability of individual chimpanzees to adjust to the metabolic conditions faced during their pregnancy instead of strict adherence to a strict developmental program. Despite this apparent flexibility in the length of gestation, we found that there were limits to viability, and those chimpanzees born before 200 days were never viable.

Advances in obstetrics, perinatology, and neonatology have enabled humans born as early as 22 weeks into gestation to survive and live productive lives [62]. However, these advances are recent, and human infants born preterm (i.e. <37 weeks) in the recent and distant past usually died. The current study suggests that chimpanzee viability without obstetric intervention is approximately 200–208 days. Advances in medicine have made viability of human preterm infants a possibility as early as 22 weeks, but this would not have been the case during most of our evolution. Remembering the discrepancies between the two species in terms of calculating conception date, it is possible to infer that the 200-day threshold for viability in chimpanzees reported in the current work is roughly equivalent to 31 weeks of human gestation. If this was the threshold of viability for infants at the time of the last common ancestor of humans and chimpanzees we can infer that chimpanzees normal gestation length is 20 days after reaching viability, but humans is approximately 50 days beyond. Thus, humans require more time than chimpanzees in the womb before they are able to pass Ellison's tipping point according to the crossover hypothesis.

This lengthening of human gestation is ironic given the anatomical constraints placed on human parturition due to the pelvic and neuroanatomical adaptations that humans have evolved. Humans have the longest gestation amongst hominids [63], therefore it is parsimonious to assume that human gestation length has increased since we last shared a common ancestor with chimpanzees. This increase can partly be explained by the increase in body size, human females weigh on average 12 kilograms more than chimpanzee females [63]. However, mountain gorilla females are larger than human females by 10–40 kilograms [63] yet their gorilla gestations are on average 26 days shorter than their human counterparts [64]. Therefore, there must have been a selective advantage that outweighed the cost of difficult parturition in the human species. Humans require a longer gestation despite the fact that they are born at a generally more altricial stage of development than chimpanzees [22]. The likely solution to this apparent evolutionary paradox is that prolonged intrauterine brain development provides an advantage that outweighs the cost of prolonged labor. Indeed, late-preterm human infants have increased relative risk for developmental delay [65], intellectual disability [66], and psychological disorders [67]. Taken together, these findings suggest that the increase in gestation length in humans may have facilitated the evolution of higher cognitive abilities. This implies that humans must cross a gestational threshold time longer than the inferred mean gestation length of the last common ancestor of humans and chimpanzees in order to reduce the risk of cognitive deficits. These insights are only possible with the current data on the mean and range of chimpanzee gestation length.

### Conclusions

This study documents pregnancy loss and normal gestation in captive chimpanzees. We documented spontaneous abortions, preterm delivery, and perinatal death in captive chimpanzees. We can conclude that the vast majority of pregnancies studied were normal, term pregnancies that resulted in healthy offspring. As in humans, pregnancy loss is not uncommon in chimpanzees, although we were not able to document pre-clinical pregnancy loss. Further work needs to be done in measuring the length of gestation and other relevant parameters in non-human primates in order to understand the evolutionary origins and consequences of preterm parturition in mammals.
